# Investigation of different LSTM-based encoder-decoder neural networks for vehicle speed prediction

**DOI:** 10.1038/s41598-025-19592-5

**Published:** 2025-09-23

**Authors:** Paul Heckelmann, Sandro Chris Breuer, Stephan Rinderknecht

**Affiliations:** 1 Department of Mechanical Engineering, Institute for Mechatronic Systems, TU Darmstadt, Darmstadt, Germany; 2 Department of Mechanics, TU Darmstadt, Darmstadt, Germany

**Keywords:** Long short-term memory, Artificial neural networks, Speed prediction, Intelligent vehicles, V2X, Traffic simulation, Mechanical engineering, Electrical and electronic engineering

## Abstract

In this work, different Long Short-Term Memory (LSTM) encoder-decoder artificial neural networks are investigated. These networks differ in their complexity. The aim of this work is to evaluate whether complex networks are necessary for vehicle speed predictions or whether simple and less computing power intensive networks can handle this task also with sufficient accuracy. For this task, simulatively generated data is used, which is created with a Simulation of Urban Mobility (sumo) traffic simulation from an urban traffic scenario from the city center of Darmstadt, Germany. The data generating process is described as well as the data handling and processing. For the investigated network architectures, grid searches are executed to investigate the sensitivity to four main hyperparameters, the mini batch size, the learning rate, the weight decay and the number of LSTM cells within each layer. The results are then evaluated based on their accuracy regarding a test data set and based on the computing power required for training. The results presented in this work indicate that also less complex models can handle the task of speed predictions and, at least for these applications, simple models should be considered in order to save computing power and, as a consequence, also energy.

## Introduction

Artificial intelligence has a wide range of application from robotic systems to self driving cars. In this paper, machine learning (ML) is used for the speed prediction of vehicles within a simulated traffic environment. This speed prediction can have different benefits for e.g. the operating strategy of different drive train configurations, safety-relevant features, the possibility of reducing the energy consumption by controlling the traffic flow or the individual vehicle speed. However, the use of Artificial Intelligence (AI) is not free of charge. One downside of these systems operating based on large amounts of data is the consumed energy during the training of the models. Strubell, Ganesh, and McCallum^1^ investigated the “substantial financial and environmental costs due to the energy required to perform this computation”^1^. They concluded that, since the neural networks and the underlying data sets increase rapidly, the focus should not only be on the accuracy of the models but also on the efficiency and the corresponding carbon footprint. The crucial role of the sensitivity to hyperparameters when training the models is emphasized. Henderson et al.^2^ suggested the systematic reporting of energy and carbon emissions metrics and use the evaluation of reinforcement algorithms with respect to their consumption as an example for a responsible and climate friendly research. In a very recent paper, Mavromatis, Katsaros, and Khan^3^ examined the influence of both model structures and hyperparameters reducing the energy consumption while trying to keep the performance.

It is obvious that, although more complex networks can lead to an improvement of machine learning predictions, the performance and environmental costs of machine learning models have to be balanced when it comes to the question of choosing model structure and hyperparameters for a certain case. In this paper, the case of speed prediction is studied using a standard Long Short-Term Memory (LSTM) encoder-decoder network. By additionally varying the size of the underlying data set, i.e. the given features, it is possible to correlate the model structures and their complexity with their performance and energy consumption. Interestingly, the increasing performance of more complex model structures investigated here does not always weigh the expense in computation time.

### Use of speed predictions

Speed predictions are already used in many different applications for Advanced Driver Assistance Systems (ADAS), powertrain operating systems and traffic management systems. For all these use cases speed predictions can have a significant benefit to improve such systems by anticipating the future state of one or multiple vehicles. As described below, the execution of the prediction models and the underlying data basis differs in every approach. The database is therefore mainly dependent on data availability in the corresponding use case. The selection of the model is particularly based on this data basis.

For example, Ye et al.^4^ used speed predictions for the operation strategy of a Hybrid Electric Vehicle (HEV). The speed prediction is used here for an optimal use of the electric machine and the combustion engine and a favorable gear shifting strategy. The operation strategy is aiming for using both engines within their combined optimal operation point. In this work, a fully connected Convolutional Neural Network (CNN) is used for the speed prediction, therefore also available Vehicle-to-everything (V2X) information is used. This approach provides for a reduction of CO_2_ emissions of 16.04% for the shown cases^4^.

Also, Guo et al.^5^ tried to use speed predictions for an enhanced operation strategy for a HEV. Here, a model predictive control approach is used for a predictive energy management system. Therefore, a radial basis function neural network is used to predict the vehicle speed based on velocity and acceleration data. The training-data is gathered from standard driving cycles, e.g. Worldwide harmonized Light vehicles Test Procedure (WLTP). With this approach, a reduction in consumption of up to 5.37% can be achieved^5^.

Next to optimizing the operation strategy of HEV, also the operation strategy of Fuel Cell Electric Vehicle (FCEV) can be improved by speed predictions. Quan et al.^6^ tried to reduce operation cost and drive train degradation with their approach. Therefore, an inflated 3D inception LSTM network is used to predict the vehicle speed. This is trained with historic vehicle speed and image sequence data. With their approach, a reduction in operation cost of 3.12% and 0.51% reduction in powertrain degradation can be expected^6^.

Also, Zhang et al.^7^ worked on an optimized operation strategy for FCEV. The goal in their work was the lowering of operation cost per hour and fuel cell degradation. In this approach, an exponential smoothing law speed prediction with Markov-based correction is used to forecast the speed of a vehicle. Therefore, only historic speed data is used in order to determine the future speed of the ego-vehicle. With this approach, a reduction of 3.74% of operation cost per hour can be determined, with costs for fuel cell degradation already being included^7^.

Next to operation strategy, there are other applications for speed prediction models. For example, it can be used to improve intelligent traffic management systems^8^ and tasks for semi or full autonomous vehicles.

Eichenlaub, Heckelmann, and Rinderknecht^9^ use a vehicle speed prediction to forecast the velocity of the ego and the leader vehicle in order to use this information for an intelligent longitudinal control. They use sensor- and V2X-data to learn an encoder-decoder LSTM based model. The database of this approach is gathered from a Simulation of Urban Mobility (sumo)^10^ simulation which is representing an urban traffic scenario. With this approach, an optimized longitudinal control of a vehicle in an urban environment can be expected. According to^11^, the reduction of energy demand for a battery electric vehicle is up to 6.8% with this approach.

The areas of applications above showed, that the accurate speed prediction of an ego-vehicle is widely used in ADAS and other intelligent vehicle systems to improve safety, reduce cost and save energy. Therefore, the efficient execution and accurate prediction results are a main focus of researchers.

Also it is getting clear that most approaches use only a small selection of features for the speed prediction task, but some approaches already try to estimate the potential with the availability of V2X information. Thus this work investigates two different data sets, one with a minimal, sensor based feature set and another one with V2X information available. This means that the models can be analyzed for both databases.

### Execution of speed predicting algorithms

In general, there are quite a lot of options how a speed prediction can be executed, but according to Lefevre et al.^12^, machine learning approaches are showing the highest potential in this task. In the past, LSTM approaches in particular have been used for this use case due to their good processing of sequences, but also other approaches have been tested^13^. In Table 1 a brief overview over different approaches for vehicle speed prediction is given.

**Table 1:** Overview of different approaches for vehicle speed prediction.

References	Network type	Features	Outcome
^14^	Bi-LSTM (3 parallel LSTMs with different lookbacks) + Attention	Speed data (1 h lookbacks) is used	Faster convergence and slightly better accuracy with attention layer
^15^	CNN pooling + LSTM encoder-decoder	Vehicle, traffic (loop detectors), weather, precipitation data is used	Comparison of faulty vs. corrected input data; real-world benchmark
^16^	4 LSTM encoder-decoder variants (LSTM, PSO-LSTM, GSA-LSTM, GSA-BiLSTM)	Including particle swarm optimization (PSO) and gravitational search algorithms (GSA)	Combination of GSA and Bi-LSTM gives highest prediction accuracy
^17^	LSTM vs. undefined other RNN	Comparison of results with same input	LSTM better, especially for short-term predictions

It becomes evident that a wide range of machine learning algorithms and network architectures have been explored for vehicle speed prediction. The trend shows a tendency towards increasing model complexity by incorporating more components to improve prediction accuracy and convergence speed. In the following, the impact of this growing complexity on energy consumption is analyzed by comparing three different LSTM-based network types. The central question addressed is whether the increased computational effort is justified by the potential performance gains of more complex network architectures.This trade-off between computational effort and predictive performance is not addressed in the reviewed studies. Therefor, the contributions of this work can be summarized as follows:

- A systematic comparison of three LSTM-based encoder-decoder architectures with different complexity levels, applied to realistic urban traffic simulation data.
- A comprehensive training pipeline including data generation, preprocessing, normalization, and sequence construction for vehicle speed prediction tasks.
- An extensive grid search over key hyperparameters to analyze their impact on prediction accuracy for all three LSTM-based encoder-decoder architectures.
- A quantitative analysis of the trade-off between model complexity, prediction performance, and energy demand.
- Demonstration that reduced-complexity models can achieve comparable accuracy at significantly lower energy cost.

## LSTM-based prediction models

As shown in the previous section, the speed prediction models are mostly executed with LSTM-cells as encoder-decoder layout. These encoder-decoder layouts have the ability to predict time series with a complex underlying database. Because driving information and traffic data can have a complex context, these type of networks provide the necessary capability to predict speed time series based on this data.

For the approaches presented in this paper, the baseline layout is at first a LSTM-encoder-layer with a predefined number of LSTM-cells which produces the encoder-state. This information is then processed in the LSTM-decoder-layer. The different decoder layouts, which are shown in the following parts, concern the data usage of the information provided by the LSTM-encoder-layer and the internal data processing within the LSTM-decoder-layer. For the following investigation, the number of LSTM cells are the same for the encoder and the decoder layer.

### Fundamentals of LSTM-cells

LSTM-based deep learning models belong to the class of RNNs, which differ from CNNs by incorporating a feedback loop. This feedback mechanism enables RNNs to maintain internal states, making them particularly suitable for processing sequential data and exhibiting dynamic behavior over time^18,19^.

**Figure 1:**
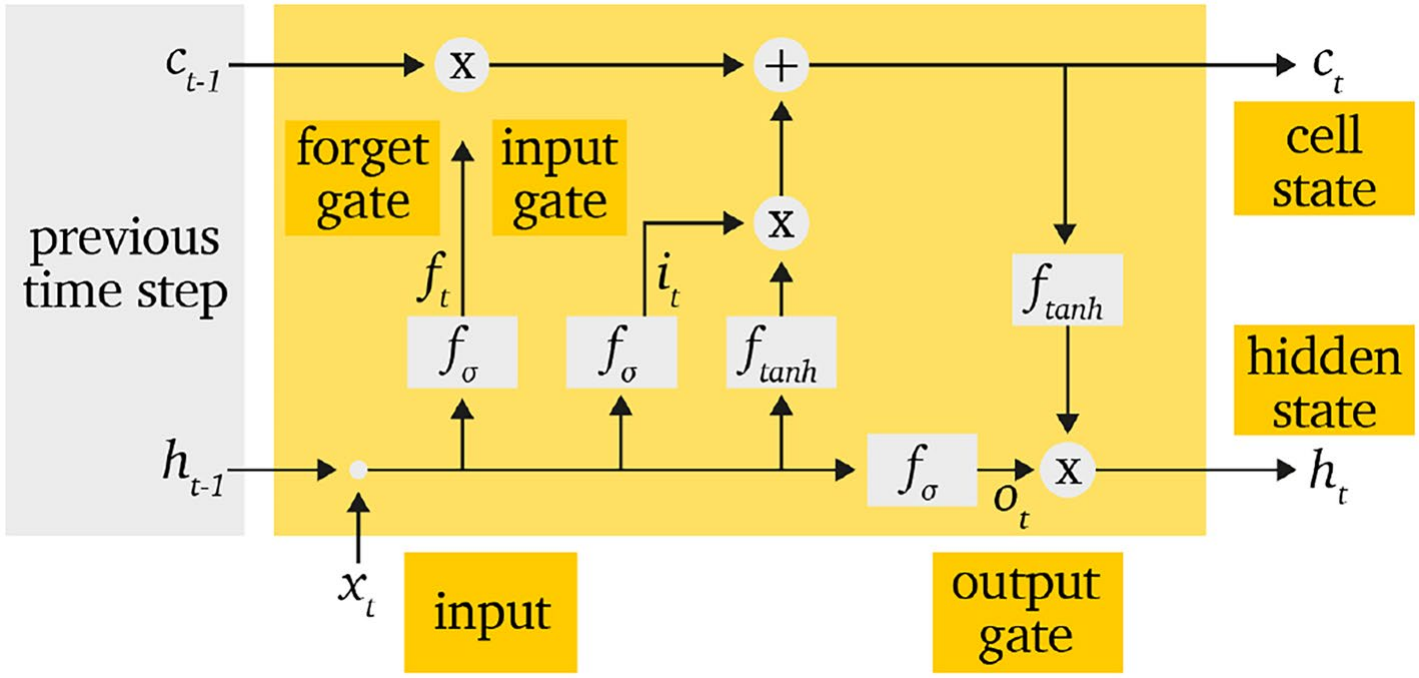
Schematic structure of data handling within a LSTM-cell according^20^.

One of the most commonly used RNN architectures is the LSTM network, which introduces three gating mechanisms: the input gate, forget gate, and output gate. These gates are interconnected through recurrent loops that control the information flow and temporal dependencies within the LSTM cell. The general structure of an LSTM cell is illustrated in Fig. 1.

The data and information flow within an LSTM cell can be described as follows:

At each time step *t* the input feature vector is denoted as $x_t$. Along with the hidden state $h_{t-1}$ from the previous time step, these inputs are used to compute the forget gate $f_t$ and the input gate $i_t$:

1 $$\begin{aligned} f_t&= \text {sig}(W_f x_t + U_f h_{t-1} + b_f) \end{aligned}$$

2 $$\begin{aligned} i_t&= \text {sig}(W_i x_t + U_i h_{t-1} + b_i) \end{aligned}$$

Here $W_f$ and $W_i$ are weight matrices for the new input $x_t$, and $U_f$ and $U_i$ are weight matrices for the recurrent connections based on the previous hidden state $h_{t-1}$. Additionally, $b_f$ and $b_i$ are bias vectors.The sigmoid activation function *sig*() is used to ensure that the gate outputs $f_t$ and $i_t$ lie between 0 and 1, thereby regulating how much information is passed through each gate.The parameters $W_f$, $W_i$,$U_f$ and $U_i$ are learned during the training process.

Based on the gate outputs and using the element-wise multiplication operator $\odot$, the cell state $c_t$ is updated as follows:

3 $$\begin{aligned} c_t = f_t \odot c_{t-1} + i_t \odot \tanh (W_c x_t + U_c h_{t-1} + b_c) \end{aligned}$$

The parameters $W_c$, $U_c$, and $b_c$ are again learned weights and biases, analogous to those in Eqs. (1) and (2).

Next, the output gate $o_t$ is computed, taking into account the current input $x_t$, the previous hidden state $h_{t-1}$, and the trained parameters $W_o$, $U_o$, and $b_o$:

4 $$\begin{aligned} o_t&= \text {sig}(W_o x_t + U_o h_{t-1} + b_o) \end{aligned}$$

The output gate determines which parts of the current cell state $c_t$ are used to update the hidden state. Based on this, the hidden state $h_t$ is calculated as:

5 $$\begin{aligned} h_t&= o_t \odot \tanh (c_t) \end{aligned}$$

During the sequence processing, both the hidden state $h_t$ and the cell state $c_t$ are passed on to the next time step, as illustrated in Fig. 1.

### LSTM-encoder-layer

**Figure 2:**
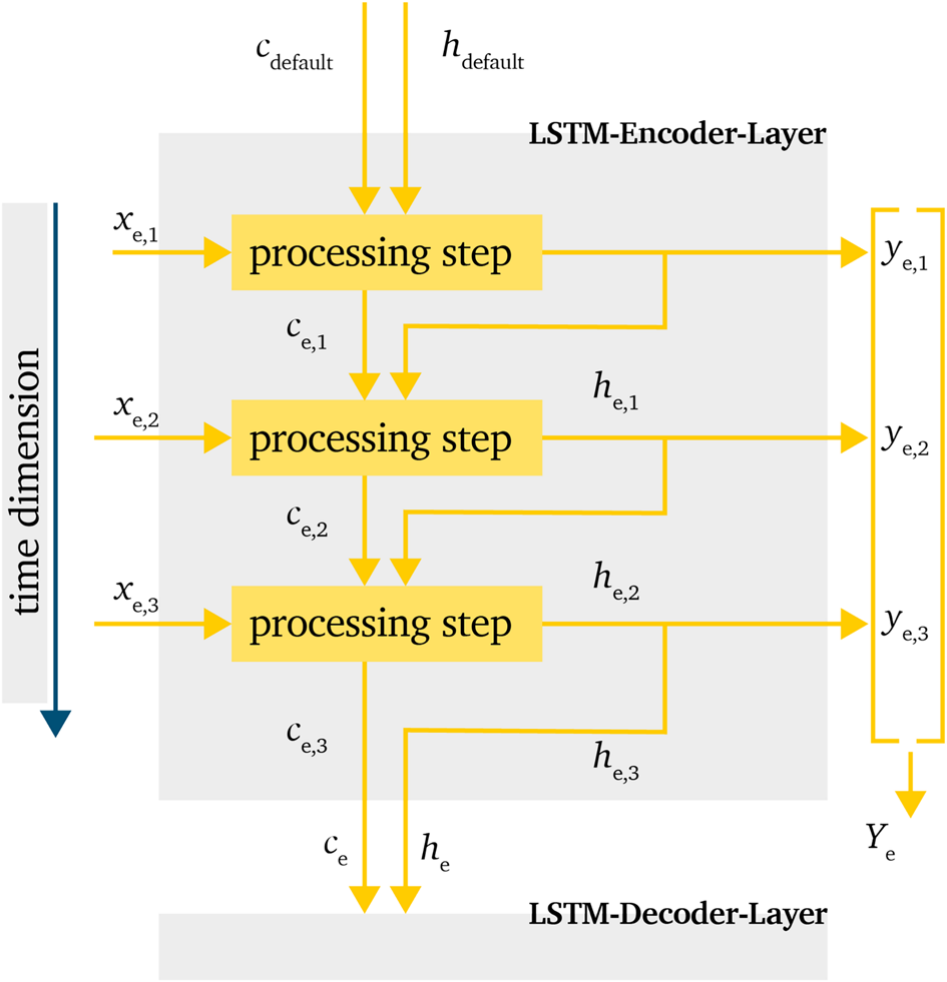
Processing of information within the LSTM-encoder-layer in a time domain representation.

The encoder-layer processes the incoming time series information as shown in Fig. 2. In this figure, the input matrix $X_e$ represents all features over the lookback time interval. Therefore $x_{e,t}$ is defined as the vector of all features for time step *t*.

For the first time step $x_{e,1}$ the LSTM-cells also receive the initial default values for $c_{\textrm{default}} = h_{\textrm{default}} = 0$.

The output of the LSTM-cells are, as mentioned in Sect. ''Fundamentals of LSTM-cells'', $c_{k,t}$, $h_{k,t}$ and $y_{k,t}$. The index *t* represents the time step within the series and *k* represents the layer of the encoder-decoder layout, in this case $k=e$ for the encoder and $k=d$ for the decoder.

In general $y_{k,t}$ is just saved as output of the corresponding LSTM-layer, $c_{k,t}$ and $h_{k,t}$ are handed over to the next time processing step $t+1$ to carry over this information. In this process, $y_{k,t}$ and $h_{k,t}$ have the same values but only $h_{k,t}$ is handed over to the next step.

At the end of the processing of the time series, the encoder state is generated as vector $Y_e$, and values $c_e$, $h_e$.

The vector $Y_e$ is not used in the further procession of the information in any of the following model layouts, so this information can be dismissed. For the further network architectures, the handling of $c_e$ and $h_e$ is part of the focus.

### Recursive model

**Figure 3:**
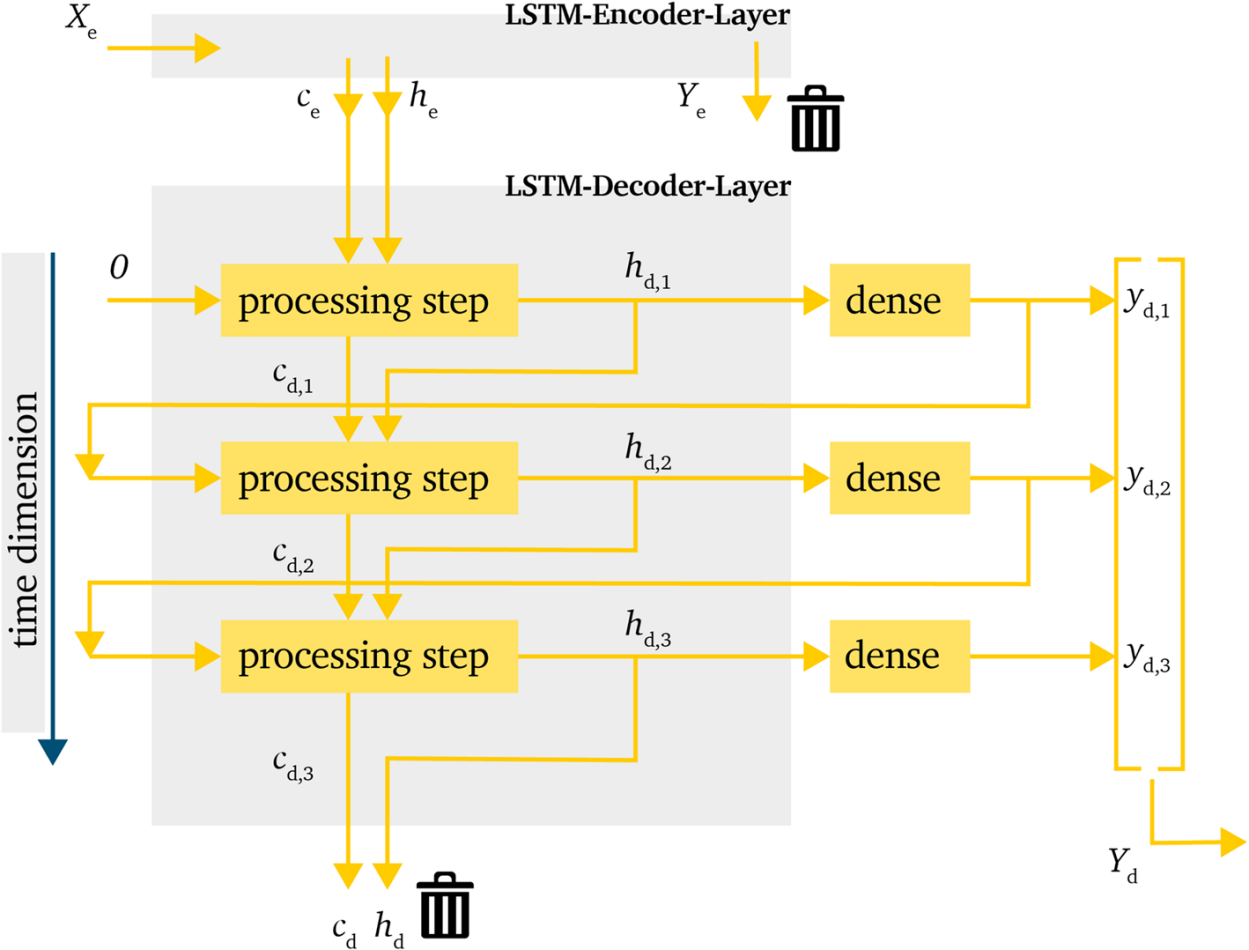
Processing of information within the LSTM-decoder-layer for the *recursive model* in a time domain representation.

The first model layout represents a recursive approach, which is commonly used within language processing. The main advantage of this model is that the input value for the decoder is based on the output of the former time step $h_{d, t-1}$. Additionally, the predicted value within a time step *t* always refers directly to the previous value $h_{d, t-1}$ by also taking into account the information of the hidden and cell state from the previous steps with $h_{d,t-1}$ and $c_{d,t-1}$. The downside of this approach is a higher risk of error propagation caused by a big deviation of the prediction in the early steps of the horizon. The data processing of this model layout is shown in Fig. 3.

In this approach, the input value of the first step within the LSTM-decoder-layer is calculated based on the initial value $x_{d,0}=0$. Additionally, within this processing step, the encoder-states $c_e$ and $h_e$ are used as initial values for $h_{d,0}$ and $c_{d,0}$.

The output values $h_{d,t}$ are then processed in a dense layer to get the final output value and shape $y_{d,t}$. The general calculation of the dense layer is executed as shown in Eq. (6).

6 $$\begin{aligned} y_{d,t} = h_{d,t}W^T + b \end{aligned}$$

The matrix $W^T$ is the transposed weight matrix and *b* is the bias vector, which are trained in advance. In the end the vector $Y_d$ represents the desired prediction vector.

### Non-recursive model

**Figure 4:**
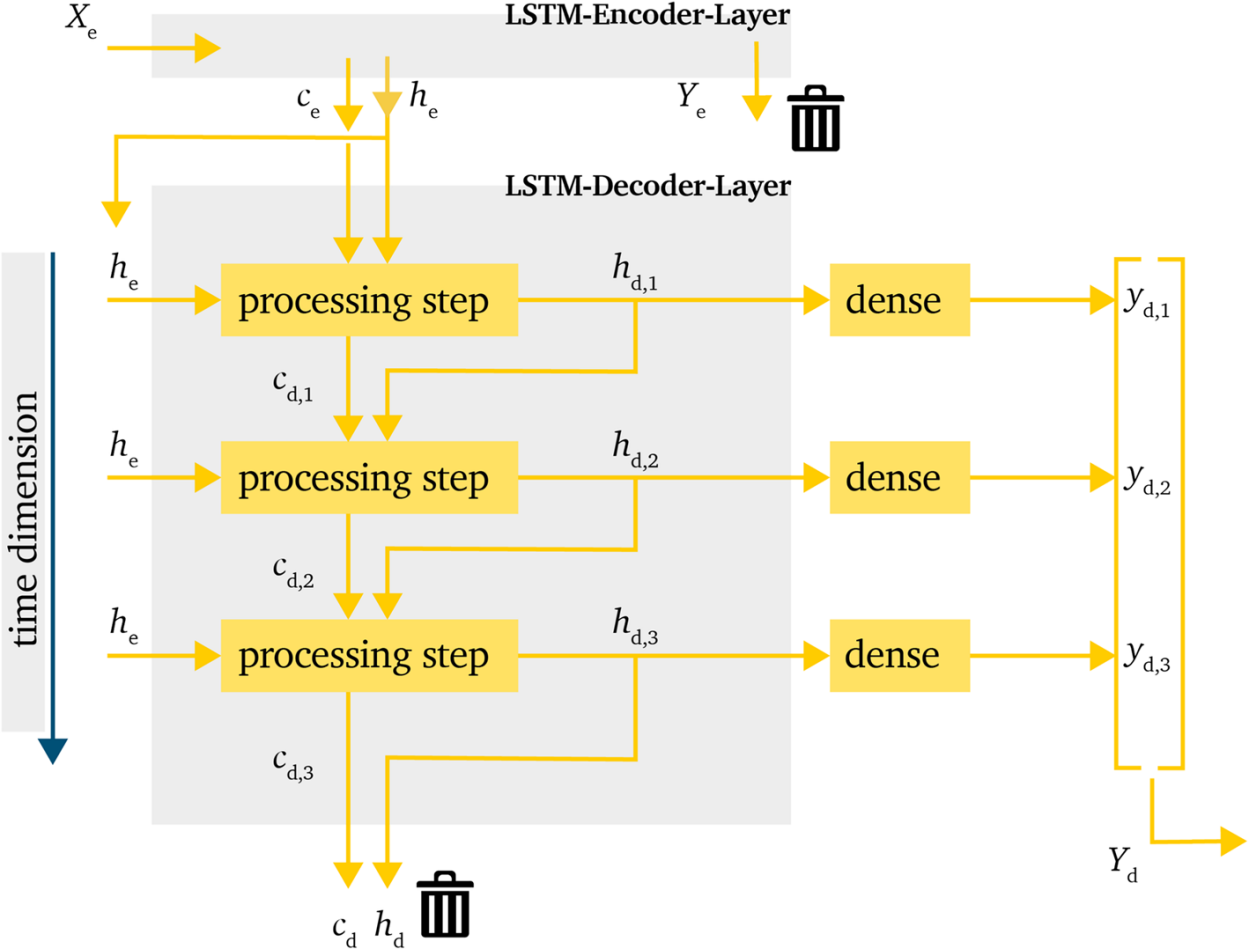
Processing of information within the LSTM-decoder-layer for the *non-recursive model* in a time domain representation.

This layout eliminates the recursive element of the model. This means that, as shown in Fig. 4, the output values $h_{d,t-1}$ are not used as decoder input of the next time step. Instead, the input is chosen to be $h_{d,t}=h_e$. In the same manner as the *recursive model* (RM) in Sect. ”Recursive model”, the *non-recursive model* (NRM) uses $c_{d,0}=c_e$ and $h_{d,0}=h_e$, and $h_{d,t}$ and $c_{d,t}$ are handed over every time step.

The advantage of this approach is that the calculation is easier due to the parallel calculation behavior. The decoder input can be generated by multiplying $h_e$, which eliminates one calculation loop. This can reduce training and prediction time. Also, the error propagation should be less relevant than for the RM due to the reference to the same input vector within every time step. But this approach also carries the risk that higher variances are not represented correctly due to the reference to the same value for every time step. Also, it is possible that higher differences from the encoder state values cannot be represented well because of to the higher influence of these values.

### Simple non-recursive model

**Figure 5:**
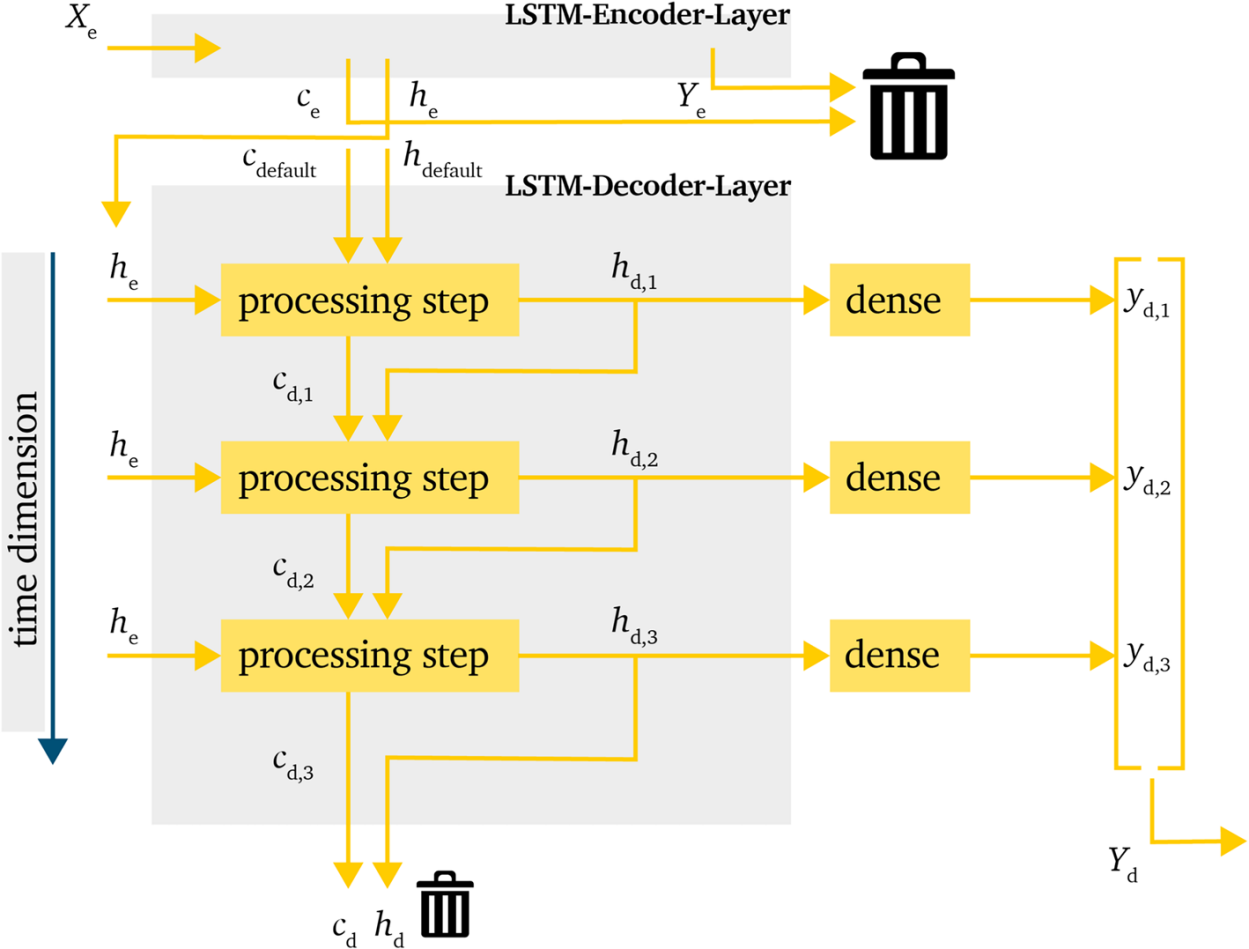
Processing of information within the LSTM-decoder-layer for the *simple non-recursive model* in a time domain representation.

The third investigated model is an even simpler approach then the NRM. The main difference here is the use of $h_e$ and $c_e$ for the LSTM-decoder-layer. The decoder-layer can be seen in Fig. 5.

Just like the NRM, the *simple non-recursive model* (SNRM) also uses the $h_e$ value from the LSTM-encoder-layer as input values for every time step. This should provide the same advantages and disadvantages as the NRM, that is lower risk of error propagation and lower variance between the predicted values within the time series as advantages and weaker correlation between the predicted values within the time series as disadvantage.

The big difference between the two models is the handling of $h_e$ and $c_e$, where $h_e$ is only used as input value as mentioned in the NRM and the value for $c_e$ is not used at all. Hence, these parameters are set to their initial default values $c_{d,0}=0$ and $h_{d,0}=0$.

Just like the NRM, the SNRM also uses a dense-layer to gather the final output value and shape $y_{d,t}$.

This adaption reduces the information handed over from the LSTM-encoder-layer to the LSTM-decoder-layer. The advantage for this model is that the prediction is less influenced by information which is gathered during the lookback horizon. This could shift the focus of the prediction more towards the future, or last, time step in the lookback horizon. The prediction can be expected to be closer to the last value and not be inflicted by continuities during the lookback horizon. A better anticipation during the first steps of the prediction horizon can be expected.

Also, due to fewer values being handed over, the time for training and prediction may be reduced.

A disadvantage is that the information of $c_e$, which represents the long term memory, is not used for the LSTM-decoder-layer and $h_e$ is solely used as decoder input but not for the initial hidden state $h_{e, 0}$. This could result in a less accurate prediction over the prediction horizon, even though an improvement can be reached in the first steps.

## Data acquisition

A main factor for the training, interpretation and discussion of machine learning based models is the underlying data basis. This paper utilizes data that has been generated through simulation. This allows the generation of any amount of training, validation and test data. Also, it allows the generation of data, which is not trackable in nowadays traffic, e.g. V2X-based information. The final advantage of using simulated generated data is the absence of errors like time delays, missing data or other data discontinuities. For this paper, the relevant data is generated with a traffic simulation within the traffic simulation platform sumo. sumo is a microscopic, inter- and multimodal, space-continuous and time-discrete traffic flow simulation platform. This means that the speed of every vehicle is modeled individually and follows the rules of a car-following-model. This car-following-model represents a real driving persons’ behavior in a traffic scenario. In addition to the car-following-model, the vehicle behavior is also affected by the lane-change-model and the models for the infrastructure, e.g. traffic lights and intersections. In this paper, the Extended Intelligent Driver Model (EIDM)^21^ is used for the car-following-model. For the other models characterizing the traffic and vehicle interaction, the default sumo models are initialized^22^.

### Generating data with SUMO

**Figure 6:**
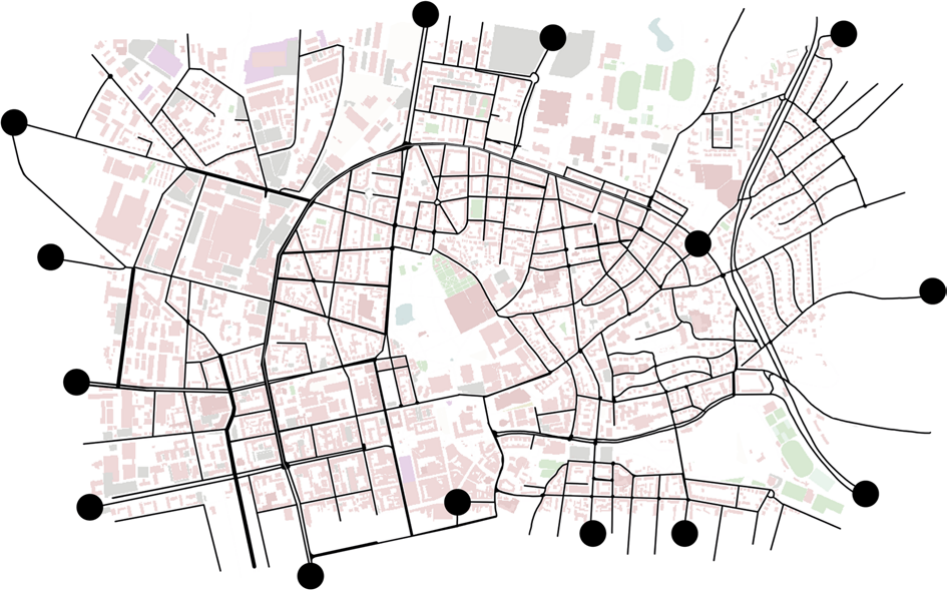
Map of the simulated area of the city center of Darmstadt with marked points with which the routes can be defined for the data acquisition. The base map was created using sumo (version 1.21.0, https://eclipse.dev/sumo/)^10^.

For this paper, the relevant training, test and validation data is generated in a validated traffic simulation of the city center of Darmstadt. This simulation is already used in^9,11^. It is divided into two separate sub-simulations, one for a rush hour traffic scenario and one for an off-peak traffic scenario. Both parts of the simulation contain approximately 1 h of simulation time. This provides enough time in order to record also longer distances within the analyzed area. The area of the city-center of Darmstadt, which is under investigation, is shown in Fig. 6. The simulation is executed with a step-size of 0.2 s.

For this paper, it is desired to cover as many traffic situations as possible occurring in the used traffic simulation. A systematic selection of routes is used to acquire data. Therefore, the points marked in Fig. 6 are connected with each other with the shortes route possible. This build the routes through the simulation area. It is assured that no points too close to each other are connected because these routes would not build representative routes. With this method, a multitude of **72** Routes are created.

To also take into account the change over time of the traffic behavior, each of these routes is executed seven times with a time distance of 500 s. Consequently, these results also take into account differences in local traffic situations.

All Routes are recorded during rush-hour and off-peak simulations, with equivalent information being included for both periods in the corresponding data.

### Data processing

The necessary information is collected from sumo with a python interface called Traffic Control Interface (traci). This interface allows a live interaction with the sumo simulation. With traci the relevant data is extracted to gather all relevant information and create the used feature set. The raw information is bundled in the preprocessing of the data collection and the features are calculated according to their definition. For this work, the same feature set as in^11^ is used. These features are shown in Table 2.

**Table 2:** All used features within this paper sorted by record types with the scaling factors, mentioned in Sect. ”Training process”.

Feature description	Data source		Scaling
	Sensor	V2X	Factor
Ego-vehicle speed	x	x	1/19.44
Leader-vehicle speed	x	x	1/19.44
Speed difference(ego & leader)	x	x	1/19.44
Ego-vehicle acceleration	x	x	1/20
Leader-vehicle acceleration	x	x	1/20
Acceleration difference (ego & leader)	x	x	1/20
Distance to leader-vehicle	x	x	1/1000
Maximum allowed speed	x	x	1/19.44
Ego-vehicle blinker signal	x	x	1
Leader-vehicle blinker signal		x	1
Mean speed on ego-vehicle lane		x	1/19.44
Local traffic density		x	4
Queue at next TLS		x	1/1000
Vehicle type leader-vehicle		x	1
Phase of next TLS		x	1
Distance to next TLS		x	1/1000
Time to next TLS phase		x	1/1000
Next lane speed limit		x	1/19.44
Distance to next speed limit change		x	1/1000
Number of lanes		x	1/5
Number of connecting routes at the next intersection		x	1/5
Distance to next intersection		x	1/1000

The shown method results in a total of 2869 km recorded training data which is equivalent to over 110 h of driving time.

## Training process

**Figure 7:**
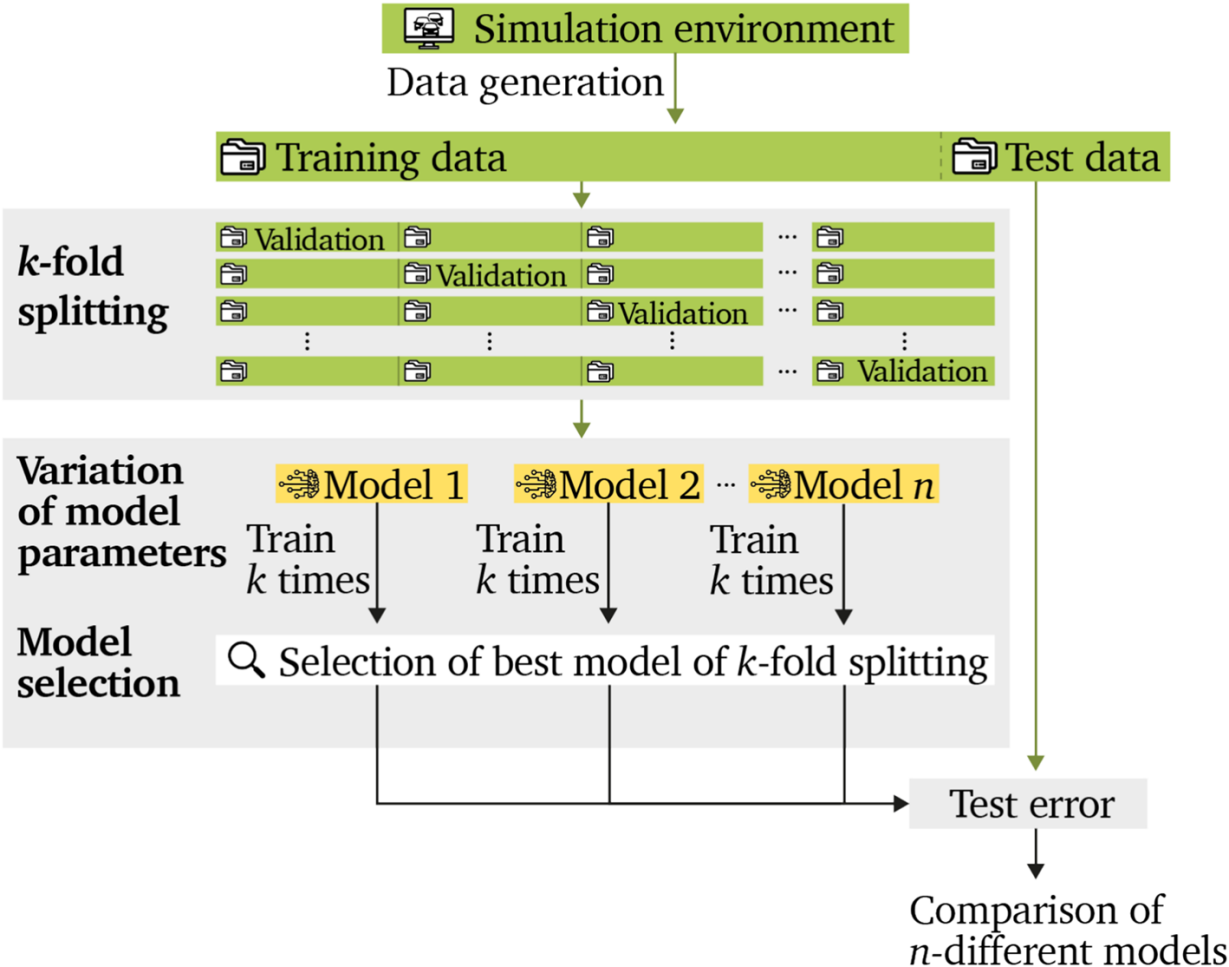
Process for the training and evaluation of the different LSTM models.

The used training method for all the models described in Sect. ”LSTM-based prediction models” is shown in Fig. 7.

As mentioned in the previous part, all relevant data is generated via sumo. The used training data is then split up into training and validation data. For excluding the deviation caused by a certain split of training and validation data, a k-fold cross validation is executed with $k=4$ folds.

With the different model layouts which contain different hyperparameter combinations, *n* different models are executed. Finally, one of the *k* folds for each of the *n* models is selected by the lowest deviation from the real values when evaluated with the test data set.

With this method, the split between training and validation data is always 75–25%.

### Data preparation

The aim of this section is to describe how raw trajectory data are preprocessed for use in the following sequence-to-sequence LSTM-based encoder-decoder architectures. All tensors are written in bold (e.g., $\textbf{X}$), sets in calligraphic script (e.g., $\mathcal {X}$), and scalars or integers in capital letters (e.g., *X*). Tensors of order *m* in $n_1 \times \ldots \times n_m$ real-valued spaces are denoted by:

7 $$\begin{aligned} \mathbb {R}\left[ n_1, ... , n_m\right] {:=} \mathbb {R}^{n_1 \times ... \times n_m} \end{aligned}$$

The data for a single route, vehicle, and traffic condition (rush hour or off-peak) is considered. These are stored in a second-order tensor $\textbf{F} \in \mathbb {R}[T, X]$, where *T* is the number of time steps and *X* the number of input features. *Y* is the number of output features.

Normalization The feature data are normalized to the interval [0, 1]. This is achieved via feature-specific scaling and offset vectors $\textbf{s}, \textbf{b} \in \mathbb {R}[X]$. The normalization is applied element-wise:

8 $$\begin{aligned} \tilde{\textbf{F}} = \textbf{S} : \textbf{F} + \textbf{B} \end{aligned}$$

where $\textbf{S}{ijkl} = \delta {ik}\delta _{jl}\textbf{s}j$ and $\textbf{B}{ij} = \textbf{b}_j$. Feature-wise min and max values are estimated across typical urban scenarios:

9 $$\begin{aligned} \textbf{s}_j&= \left( \textbf{f}_j^{\textrm{max}} - \textbf{f}_j^{\textrm{min}}\right) ^{-1} \end{aligned}$$

10 $$\begin{aligned} \textbf{b}_j&= -\textbf{f}_j^{\textrm{min}} \cdot \textbf{s}_j \end{aligned}$$

An offset is only assigned to accelerations since their minimum does not vanish:

11 $$\begin{aligned} \textbf{b}_j&= 0.5 \ \forall j \in \mathcal {J} \end{aligned}$$

12 $$\begin{aligned} \textbf{b}_j&= 0.0 \ \forall j \notin \mathcal {J}\end{aligned}$$

13 $$\begin{aligned} \mathcal {J}&= \{\mathrm {index(ego-vehicle \ acceleration)}, \nonumber \\&\mathrm {index(leader-vehicle \ acceleration)},\nonumber \\&\mathrm {index(acceleration \ difference)}\} \end{aligned}$$

with $\mathcal {J}$ being the set of acceleration-related feature indices. The results for the scaling factors are given in Table 2.

Sequence construction From $\tilde{\textbf{F}}$, input and output sequences are extracted with:

14 $$\begin{aligned} \textbf{X} \in \mathbb {R}[N, L, X], \quad \textbf{Y} \in \mathbb {R}[N, H, Y] \end{aligned}$$

where *L* is the lookback and *H* the prediction horizon. In this work, $L = H = 25$, corresponding to a 10, s horizon. With a simulation step size of 0.2, s, input/output intervals of 0.4, s was chosen. The following time index sets are defined for a given simulation time *t*:

15 $$\begin{aligned} \mathcal {L}(t)&= \{t - 2l : l \in \{0, \ldots , L-1\}\} \end{aligned}$$

16 $$\begin{aligned} \mathcal {H}(t)&= \{t + 2h : h \in \{1, \ldots , H\}\} \end{aligned}$$

To ensure that these sets remain within the simulation time bounds, the set of all time steps $\mathcal {T}$ is defined and extract only valid steps:

17 $$\begin{aligned} \mathcal {T}&= \{0, 1, \ldots , T{-}1\} \end{aligned}$$

18 $$\begin{aligned} \tilde{\mathcal {T}}&= \{t \in \mathcal {T} \mid \mathcal {L}(t) \subset \mathcal {T} \wedge \mathcal {H}(t) \subset \mathcal {T}\} \end{aligned}$$

From this, a sample-wise indexing set is introduced:

19 $$\begin{aligned} \mathcal {N}&= \{0, \ldots , N{-}1\} = \tilde{\mathcal {T}} - 2(L{-}1) \end{aligned}$$

20 $$\begin{aligned} N&= |\mathcal {N}| = |\tilde{\mathcal {T}}| = T - 2(L{-}1) - 2H \end{aligned}$$

21 $$\begin{aligned} n&\in \mathcal {N} \Leftrightarrow t = n + 2(L{-}1) \in \tilde{\mathcal {T}} \end{aligned}$$

This ensures that the corresponding input and output sequences for each sample index *n* are well-defined and lie within valid simulation time steps. With this, the training samples can be extracted as:

22 $$\begin{aligned} \textbf{X}_{n}&= \tilde{\textbf{F}}_{\mathcal {L}(t), \mathcal {X}} \end{aligned}$$

23 $$\begin{aligned} \textbf{Y}_{n}&= \tilde{\textbf{F}}_{\mathcal {H}(t), \mathcal {Y}} \end{aligned}$$

where $\mathcal {X}$ and $\mathcal {Y}$ denote the selected input and output feature indices with $|\mathcal {X}| = X$ and $|\mathcal {Y}| = Y$.

Multi-scenario aggregation For both rush hour and off-peak traffic scenarios, the above process is repeated for all route indices *R* and vehicle indices *V*, yielding:

24 $$\begin{aligned} \hat{\textbf{X}}&\in \mathbb {R}[2, R, V, N, L, X] \end{aligned}$$

25 $$\begin{aligned} \hat{\textbf{Y}}&\in \mathbb {R}[2, R, V, N, H, Y] \end{aligned}$$

Cross-validation and batching A k-fold cross-validation is applied. This can be seen in Fig. 7 splitting the data into:

26 $$\begin{aligned} \hat{\textbf{X}}&\in \mathbb {R}[k, 2, R_k, V, N, L, X] \end{aligned}$$

27 $$\begin{aligned} \hat{\textbf{Y}}&\in \mathbb {R}[k, 2, R_k, V, N, H, Y] \end{aligned}$$

Each fold is once used for validation, the others for training: and validation tensors are obtain:

28 $$\begin{aligned} \hat{\textbf{X}}^{\textrm{train}}&\in \mathbb {R}\left[ k, (k-1), 2, R_k, V, N, L, X\right] \end{aligned}$$

29 $$\begin{aligned} \hat{\textbf{Y}}^{\textrm{train}}&\in \mathbb {R}\left[ k, (k-1), 2, R_k, V, N, H, Y\right] \end{aligned}$$

30 $$\begin{aligned} \hat{\textbf{X}}^{\textrm{val}}&\in \mathbb {R}\left[ k, 1, 2, R_k, V, N, L, X\right] \end{aligned}$$

31 $$\begin{aligned} \hat{\textbf{Y}}^{\textrm{val}}&\in \mathbb {R}\left[ k, 1, 2, R_k, V, N, H, Y\right] \end{aligned}$$

For efficient training, tensors are flattened across all relevant axes The batching is performed along all dimensions excluding the fold, time and feature dimension. The total number of samples for training and validation are:

32 $$\begin{aligned} N_{\textrm{train}}&= (k-1) \cdot 2 \cdot R_k \cdot V \cdot N \end{aligned}$$

33 $$\begin{aligned} N_{\textrm{val}}&= 2 \cdot R_k \cdot V \cdot N \end{aligned}$$

Final tensors passed to the model are:

34 $$\begin{aligned} \bar{\textbf{X}}^{\textrm{train}}&\in \mathbb {R}\left[ k, N_{\textrm{train}}, L, X\right] \end{aligned}$$

35 $$\begin{aligned} \bar{\textbf{Y}}^{\textrm{train}}&\in \mathbb {R}\left[ k, N_{\textrm{train}}, H, Y\right] \end{aligned}$$

Analogously, tensors for validation and testing are constructed.

This yields well-structured, normalized, and time-aligned data sets for each fold, ready for model training. The training process is described in the next section.

### Training process

The training process is implemented in a pytorch^23^ framework. The training, validation and test tensors from Eqs. (34) and (35) are the basis for the model. All parameter, that do not appear in the grid search are shown in Table 3. All other parameters are set to the default values of pytorch^23^ version 1.12.1.

**Table 3:** Parameters and values for the LSTM models that are not set to the default values.

	Parameter	Value
Fixed	$\Delta t$	0.4 s
	Lookback horizon L	25 steps/10 s
	Prediction horizon H	25 steps/10 s
	Epochs	100
	k-fold number of splits	4
	Loss function	MAE
	Optimizer	adam
Grid	mini-batch size	$2^{12},2^{14}$
	Learning rate	$10^{-4}, 10^{-3}, 10^{-2}$
	LSTM size	16, 32, 64, 128, 256
	Weight decay	$0, 10^{-4}, 10^{-2}$

For all *k* folds, a complete model training is performed as explained in following. In each epoch, all of the given training and validation samples in the second dimension of Eqs. (34) and (35) are processed. Therefore, all of the samples are split into mini batches of mini batch size *B*. The training process is looped over all batches of the current epoch. With the index set $\mathcal {B}$ containing all the indices that extract the current batch from the second dimension ($|\mathcal {B}| = B$), the current input and output batch ($k^* \in \{1,..., k\}$) is obtained:

36 $$\begin{aligned} \textbf{x} = \bar{\textbf{X}}^{\textrm{train}}_{k^*, \mathcal {B}, \cdot , \cdot } \end{aligned}$$

37 $$\begin{aligned} \textbf{y} = \bar{\textbf{Y}}^{\textrm{train}}_{k^*, \mathcal {B}, \cdot , \cdot } \end{aligned}$$

The input batch is then fed into the model and the prediction is returned. For the evaluation of the deviation, the *loss function* (also called criterion) comes into place and calculates the loss from the output batch and its prediction. Then the backward propagation is performed, the gradients are clipped and the optimizer performs the step.

After this is done for all batches, the values of the loss function are averaged over all batches and the result is saved for the current epoch.

In a second step, the validation loss is calculated in the same manner as the training loss but without backward propagation. For this, the weights and gradients remain unchanged. This complete process of training and validation is repeated for the given number of epochs.

For an independent evaluation, the model is applied on the test data set once. The process is exactly the same as within the validation loop before.

With this method, the model and its training information for every fold of the current parameters can be obtained. In order to be able to examine the change of the dependency of the model behavior on the different parameters, a grid search is executed. The relevant parameter are varied and a model for each parameter combination can be trained. The different values used for each parameter within the grid can also be seen in Table 3.

### Examination method

**Figure 8:**
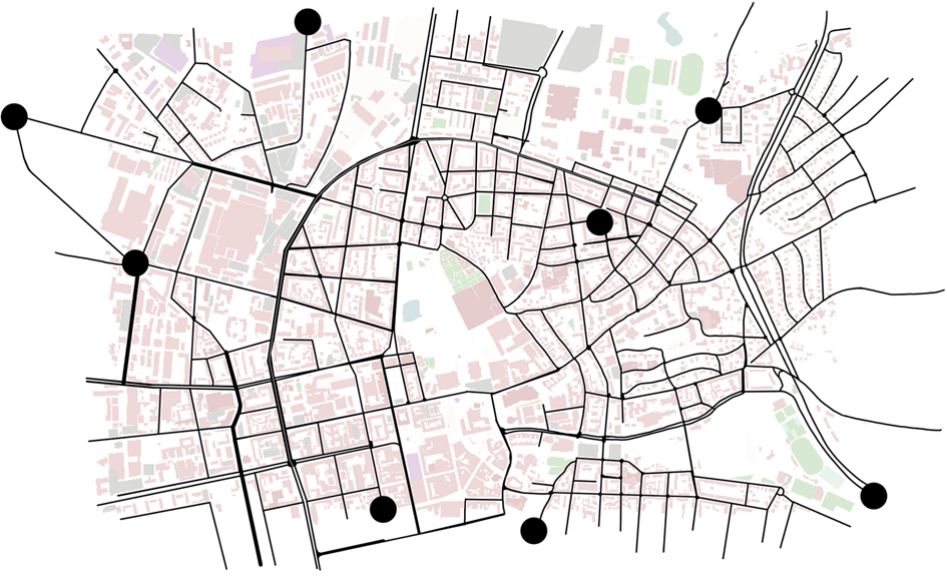
Map of the simulated area of the city center of Darmstadt with marked points with which the routes can be defined for the test data acquisition. The base map was created using sumo (version 1.21.0, https://eclipse.dev/sumo/)^10^.

As mentioned in Sect. ''Comparison of differnet layouts'', the comparison of the models is made by an independent test data-set, which is generated by an similar method as the training data but by using the start and end points shown in Fig. 8. This data set contains approximately 480 km of driven data.

To compare the models, the Mean Absolute Error (MAE) is used. This is calculated by Eq. (38), where value $\hat{v}_i$ is the predicted value and $v_i$ is the real value.

38 $$\begin{aligned} \text {MAE} = \frac{1}{n} \sum _{i=1}^{n} \left| \hat{v}_i - v_i \right| \end{aligned}$$

For the following investigation, the MAE is evaluated for the whole prediction horizon combined, where the distribution within the sequence is neglected, but also the MAE for each time step within the horizon is investigated in order to evaluate the behavior of the models in this capacity.

Another method to investigate machine learning models is the evaluation of the learning curves, so as to examine the general training behavior and whether overfitting occurs at a certain point. Within these curves, the training and validation loss is presented. The deposit loss function is also the MAE.

Moreover, the training time per epoch is evaluated. This value is very dependent on the used hardware and utilization of the hardware. For that reason, for all the following results, the same hardware, a NVIDIA RTX A5000 Graphics Processing Unit (GPU) was used and an equal, high utilization can be assumed. According to the manufacturer, the power under maximal use is measured to be 250 W^24^. With this information and the number of epochs, the energy consumption for the training process can be extrapolated. Even though other parts of the hardware have an influence on the total energy consumption and also the data preprocessing is not involved here, the training with the GPU should be the main part of energy consumption within the training of the model. Therefore, the energy consumption can be assumed to be directly proportional to the training time per epoch, which is displayed in the following results. With higher costs for energy and also the need to save energy for environmental reasons, this value also factors in to choose a suitable parameter combination for a prediction model.

## Identification of not relevant parameters

In the first step, the parameters that don’t need to be considered in the final grid search are identified. This reduces the number of parameter combinations and hence simplifies the examination and evaluation of the results.

### Mini batch size

**Figure 9:**
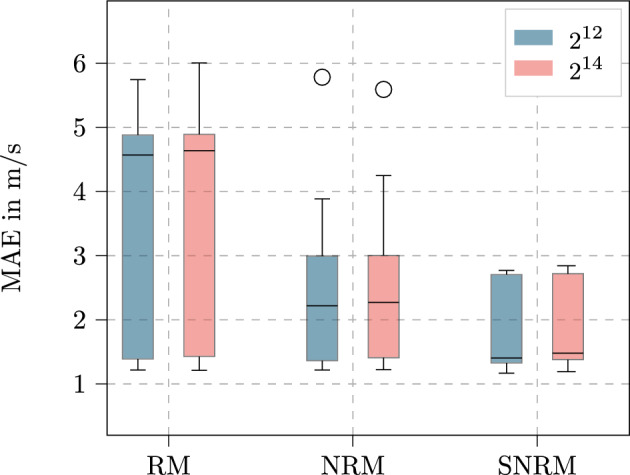
MAE for both investigated mini batch sizes of $\mathbf {2^{12}}$ and $\mathbf {2^{14}}$ and all model layouts for the sensor feature set. The boxplot shows the median as a line, the interquartile range as the box, which contains the middle 50% of the data, and whiskers extending to 1.5 times the interquartile range. Outliers are shown as individual points.

The first parameter which can be identified for this is the mini batch size. As you can see in Fig. 9, the variation of the mini batch sizes shows very similar results for all models. In this figure, all parameter combinations with the corresponding mini batch size are displayed in a box plot for the different model layouts that are shown across the abscissa. Exemplary, the results for the sensor feature set are shown.

Even though the different models show different behaviors, which will be discussed in detail in the following sections, the influence of the mini batch size can be assumed to be negligible. This statement only applies to the values described here, it can be assumed that the consideration of the mini batch size is still relevant for larger and smaller values.

Therefore, for the following, the mini batch size of 2^12^ is used, due to the lower need for memory space and the faster processing time.

### Weight decay

**Figure 10:**
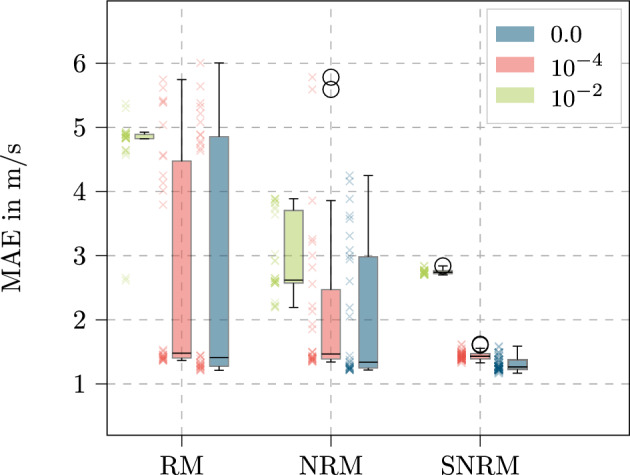
MAE for all three investigated weight decays of $\mathbf {0.0}$, $\mathbf {10^{-2}}$ and $\mathbf {10^{-4}}$ for all model layouts with the sensor feature set. The boxplot shows the median as a line, the interquartile range as the box, which contains the middle 50% of the data, and whiskers extending to 1.5 times the interquartile range. Outliers are shown as individual points.

The second parameter which is investigated in this part is the weight decay. The results of the different weight decay parameters for the three model layouts is shown in Fig. 10 exemplary with the sensor based data set.

In this figure, the box plots represent all models for the corresponding weight decay and model layout. To understand the partly large areas within the quartiles, the scatter of the underlying results are displayed as well.

These results show for all model layouts that the lowest MAE values occur for a vanishing weight decay. However, especially for the RM, this conclusion is not justified by the box plots solely. Nonetheless, with the scatter representation, two clusters can be perceived, one with an MAE over 4m/s and one cluster under 2m/s. As it is investigated in detail in Sect. ”Results - grid search”, the cluster with high MAE values follows from small numbers of LSTM cells within the layers. Even though this is not as clear for the NRM as it is for the RM, this also applies to a significant extent for this model

Mitigating the significant impact of these low LSTM cell numbers would therefore result in even clearer representation of the weight decay results.

**Figure 11:**
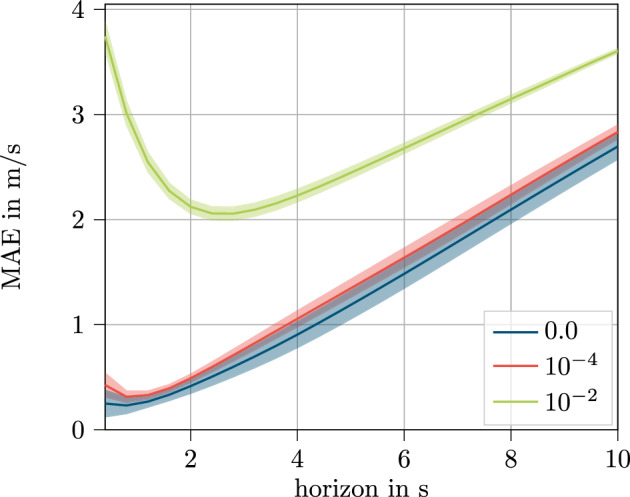
Results across the predicted horizon for the sensor feature set for weight decay values of $\mathbf {0.0}$, $\mathbf {10^{-2}}$ and $\mathbf {10^{-4}}$ with standard deviation.

A significant increase in MAE for the weight decay of 10^−4^ is apparent. In order to investigate the reason for that, the MAE values across the horizon are displayed in Fig. 11. For this, the SNRM is used representatively with the sensor data set.

In Fig. 11, the mean MAE value for the prediction for the given time step within the prediction horizon is displayed with all model parameters being included. Additionally, for the purpose of showing the deviation of the results for the different parameter combinations, the region of one standard deviation above and underneath the mean value is shown around the curves.

This underlines the results from Fig. 10, where weight decay values of 10^−2^ predict speed with notably poorer accuracy than the other investigated values of 10^−4^ and 0.0. It becomes obvious that the weight decay has a major influence on the prediction performance of the first time steps within the horizon. Towards the end of the prediction horizon, the mean MAE curves approach.Nevertheless, an offset is still visible.

The impact on the first time steps within the prediction horizon can also be seen for weight decay of 10^−4^ to a small extent. This is why only the weight decay of 0.0 is used in the following work.

## Results - grid search

After the exclusion of two parameters, the number of parameter combinations for each model is reduced from 90 to 15. In the following part, these combinations are evaluated for each model layout and for both the sensor based data set and the V2X based data set. The model parameter combinations are evaluated with respect to the MAE and the training time per epoch.

### Recursive model

In Table 4, the results for the RM are displayed. In general the best model for both data sets can be found with a learning rate of 10^−2^ and a LSTM cell number of 128, but all models with a LSTM cell number above 64 show similar results regarding the test MAE. For models with a lower LSTM cell number, the error increases significantly.

**Table 4:** Results of the recursive-model with test MAE and calculation time for one training epoch, sorted w.r.t. V2X data test MAE.

Model param.		V2X data		Sensor data	
LSTM cell no.	Learning rate	Test MAE in m/s	Time per epoch in s	Test MAE in m/s	Time per epoch in s
128	10^−2^	0.808	35.06	1.216	35.11
**258**	$\varvec{10^{-2}}$	**0.811**	**10.03**	**1.229**	**9.97**
64	10^−2^	0.816	9.01	1.234	8.95
128	10^−3^	0.901	34.93	1.282	34.95
64	10^−3^	0.926	9.11	1.273	9.05
258	10^−3^	0.946	10.02	1.264	10.06
258	10^−4^	0.998	10.09	1.336	10.09
128	10^−4^	1.022	34.99	1.324	35.11
64	10^−4^	1.057	9.11	1.389	9.06
32	10^−2^	4.636	5.37	4.748	5.11
16	10^−2^	4.710	5.34	4.673	5.00
16	10^−4^	5.304	5.49	4.880	5.18
16	10^−3^	5.408	5.54	4.889	5.13
32	10^−3^	5.432	5.50	5.639	5.30
32	10^−4^	5.515	5.61	5.324	5.31

Taking a look at the training time, it becomes obvious that the the correlation between the training time per epoch and the number of included features is negligible.

Furthermore, there is a trend that higher LSTM cell numbers result in higher training times. An exception to this are the model parameter sets with an LSTM cell number of 128. Those seem to require a particularly long training period of about three times of the models with LSTM cell number of 258. This can be explained by an unfavourable interaction between the LSTM cells for this model parameters.

Due to the small difference in test MAE between the best and second-best model, which are the same for both sensor based and V2X based data, and the significant improvement of training time of the second-best model, the second-best model is used representatively for this model layout. This parameter combination is written in bold within the table.

### Non-recursive model

In Table 5, the results for the NRM are presented in the same way as the RM in the previous part.

**Table 5:** Results of the non-recursive-model with test MAE and calculation time for one training epoch, sorted w.r.t. V2X data test MAE.

Model param.		V2X data		Sensor data	
LSTM cell no.	Learning rate	Test MAE in m/s	Time per epoch in s	Test MAE in m/s	Time per epoch in s
258	10^−2^	0.774	11.27	1.217	11.20
128	10^−2^	0.788	9.74	1.226	9.69
**64**	$\varvec{10^{-2}}$	**0.810**	**3.35**	**1.219**	**3.32**
258	10^−3^	0.886	11.27	1.268	11.23
64	10^−3^	0.902	3.43	1.244	3.36
128	10^−3^	0.908	9.77	1.237	9.70
128	10^−4^	0.933	9.81	1.300	9.81
258	10^−4^	0.941	11.32	1.265	11.30
64	10^−4^	1.040	3.47	1.336	3.42
16	10^−4^	3.012	2.32	3.575	2.16
32	10^−3^	3.303	2.28	3.086	2.20
16	10^−3^	3.430	2.25	2.945	2.12
32	10^−4^	3.662	2.43	3.884	2.36
32	10^−2^	3.706	2.23	2.190	2.13
16	10^−2^	4.001	2.13	2.994	2.02

The NRM also shows a drop in the test MAE for LSTM cell numbers under 32. Also all the other model parameter combinations show very similar MAE results. But in comparison to the results in Sect. ”Recursive model”, the drop is only about three times as high here. This could be an indicator that this model layout can handle lower LSTM cell numbers better than the RM.

For this model, both feature sets have the best test MAE again for the same parameter combination at 258 LSTM cells and a learning rate of 10^−2^.

Regarding the training time for the NRM, there is still no significant difference between the smaller and the bigger feature set. The training time per epoch steadily increases with the number of LSTM cells. A significant increase is especially displayed between 64 and 128 LSTM cells. A doubling of the LSTM cells is here accompanied by a tripling of the training time per epoch.

Because of the small differences between the test MAE values and the significant lower training time per epoch, for this model the third best model for V2X data and second best for sensor data is chosen for further evaluations. This parameter set is also written in bold within Table 5.

### Simple non-recursive model

In Table 6, the results for the SNRM are displayed. Also for this model layout, the model with an LSTM cell number of 258 and a learning rate of 10^−2^ provides the lowest test MAE value for both feature sets.

**Table 6:** Results of the simple non-recursive-model with test MAE and calculation time for one training epoch, sorted w.r.t. V2X data test MAE.

Model param.		V2X data		Sensor data	
LSTM cell no.	Learning rate	Test MAE in m/s	Time per epoch in s	Test MAE in m/s	Time per epoch in s
258	10^−2^	0.802	11.25	1.212	11.15
128	10^−2^	0.812	9.72	1.223	9.67
**64**	$\varvec{10^{-2}}$	**0.823**	**3.35**	1.216	3.29
128	10^−3^	0.872	9.73	1.261	9.73
64	10^−3^	0.872	3.43	1.231	3.32
258	10^−3^	0.891	11.25	1.224	11.20
128	10^−4^	0.945	9.80	1.294	9.80
258	10^−4^	0.946	11.29	1.273	11.28
64	10^−4^	0.985	3.46	1.371	3.33
32	10^−2^	2.286	2.21	1.168	2.14
32	10^−3^	2.409	2.31	1.231	2.19
16	10^−4^	2.445	2.27	1.489	2.14
***16***	${\varvec{10}}^{{\varvec{-2}}}$	2.507	2.08	***1.222***	***1.98***
16	10^−3^	2.687	2.21	1.326	2.08
32	10^−4^	2.776	2.41	1.485	2.25

Just like in the previous results of Sect. ”Non-recursive model”, the best and the subsequent results do not differ a lot, but the increase in the test MAE for LSTM cell numbers under 32 is only apparent for the V2X feature set. This is a major difference to the other displayed models. For the sensor based feature set, the test MAE results are all within a range of around 0.2 m/s. But also for the V2X based dataset, the test MAE approximately only doubles after the drop-off, which is a lower margin than for the other model layouts. This yields a lower impact by the LSTM cell number compared to the other model layouts. It can be assumed that this model layout can handle lower LSTM cell numbers even better if the feature set represents a smaller number of features.

Regarding the training time per epoch, the results are very similar to the results in Sect. ”Non-recursive model”, hence the training time per epoch increases for a larger number of LSTM cells. For most parameter combinations, the difference between the two models in Sects. ”Non-recursive model and ”Simple non-recursive model” for the same parameter combination is just a few hundredths of a second.

Due to the different test MAE values for lower LSTM cell numbers, two different parameter combinations are chosen for further investigation of this model layout. For the V2X feature set, the model with a LSTM cell number of 64 and a learning rate of 10^−2^ is chosen. This model is chosen due to its good test MAE value which differs from the best test MAE by only around 0.02 ms, but also has a more than three times lower training time per epoch. This model is written in bold within the table above.

For the sensor feature set, a different model parameter set is chosen. Here, the model with 16 LSTM cells and a learning rate of 10^−2^ is selected. This is selected because the test MAE only differs by 0.01 ms from the best parameter combination but has a more than 400% faster training time per epoch. The slightly poorer predictive ability is relativized by the significantly faster computing time and thus the significantly lower energy consumption. Therefore, the model which is written in bolditalic is used for further evaluation with the sensor data set.

## Comparison of differnet layouts

After individually examining the impact of the hyperparameters for each model layout thus far, the model layouts are compared with each other for a better understanding of the impact of the different data handling mechanisms. This is done for each feature set individually. The investigated models are highlighted in the corresponding tables in Sect. ”Results - grid search”. Additional to the comprehensive test MAE values in the tables, also the test MAE over the predicted horizon and the loss curve for training and validation is evaluated in the following part.

### V2X feature set

**Figure 12:**
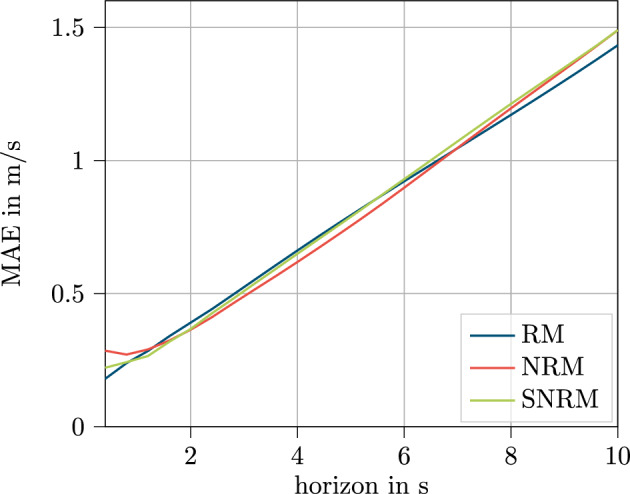
Results for the test data set displayed over the predicted horizon for the V2X feature set.

In Fig. 12, the deviation is shown across the predicted horizon for the three models considered.

The curves for all three models follow a very similar pattern and only small differences can be seen. Solely, the test results for the first and the last time steps show some slightly different behavior. One can see that the test MAE for the RM increases steadily, having the lowest MAE in the beginning for all models. Also, the SNRM has a steadily increasing MAE, but the curve is noticeably flatter for the initial steps compared to the RM. The NRM shows the best MAE for the second time step at 0.8 s, before having a steady increase in MAE for the following time steps.

Throughout the horizon, the performance of the models alternates and follows a similar pattern until the RM shows better results compared to the other models towards the last time steps. This behavior shows that error propagation seems to be less problematic for the RM than previously assumed. Also the partially not transferred information for the SNRM seems not have to an as significant impact as assumed, at least for the V2X feature set.

**Figure 13:**
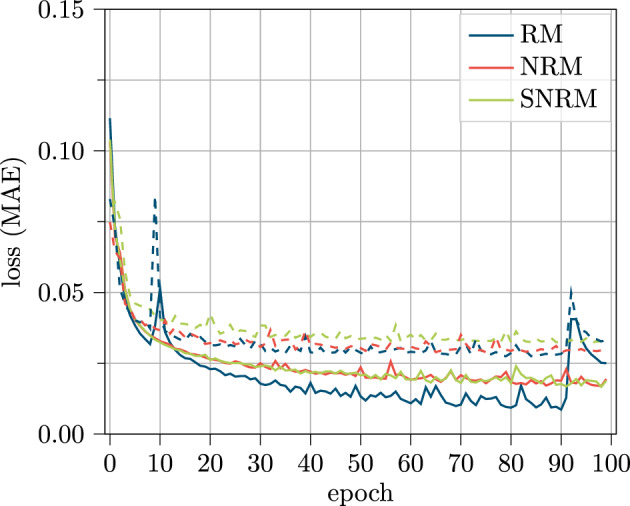
Loss curve for training (solid) and validation (dashed) data over the training process of 100 epochs for the V2X based feature set.

In Fig. 13, the loss curves for the training and for the validation dataset are presented. Even though the training curve of the RM has lower MAE values until epoch 90, the validation curve does not differ a lot from the curves of the NRM and SNRM.

The most obvious differences between the curves are the occurring peaks within the training of the RM. This may be due to a too high learning rate but will be examined in detail in the following section.

In general, all curves show a reasonable and similar learning behavior and overfitting is not present. From the course of the curves, it can be inferred that a higher epoch number could result in even better prediction results, even though this is not investigated for this work.

### Sensor feature set

**Figure 14:**
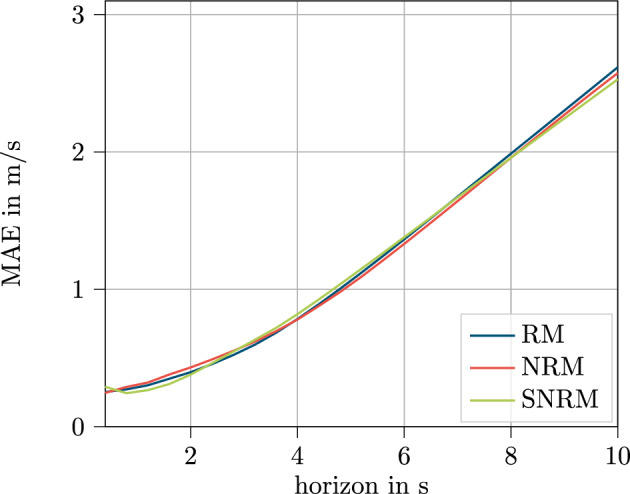
Results for the test data set displayed over the predicted horizon for the sensor feature set.

In Fig. 14, the results for all models with the sensor data set are displayed over the horizon.

The curves for the different models are even closer together and exhibit an even more similar trend than in the V2X dataset. Only tiny differences can be seen, that is the SNRM again shows the best MAE values for the second time step at 0.8 s. Both other curves increase steadily along the prediction horizon. These results reaffirm the similar results within the tables in Sect. ”Results - grid search”.

**Figure 15:**
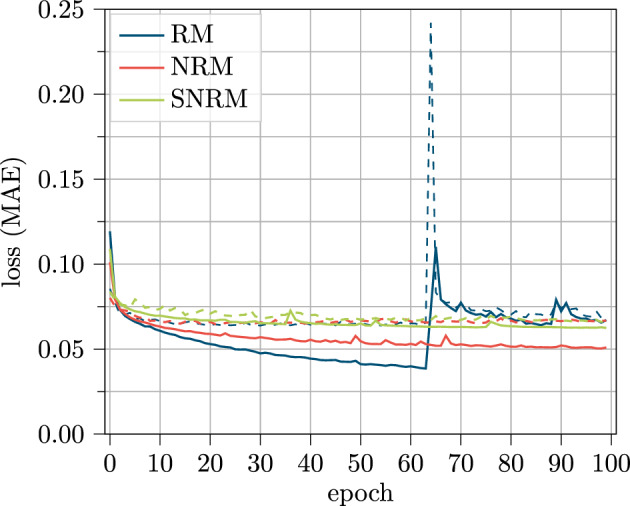
Loss curve for training (solid) and validation (dashed) data over the training process of 100 epochs for the sensor based feature set.

These results indicate that for the sensor feature set, the model data handling is not relevant and all models can handle these input information similarly when compared with respect to the best parameter configuration. For further investigation, the learning curve for this dataset is investigated in Fig. 15.

Here, the learning behavior is pretty similar to the behavior of the V2X dataset in Fig. 13. Again, the curve of the RM has a lower training loss until epoch 60, but the validation loss does not differ from the other models. In contrast to the V2X feature set, the NRM and SNRM differ for the training loss but also have similar validation losses. As well in this figure, the plots show no sign of overfitting and slightly better results with more trained epochs can be assumed.

Again, the RM curve shows a peak, this time at epoch 64 which is followed by significant higher training losses and a too high learning rate can be assumed for this model.

**Figure 16:**
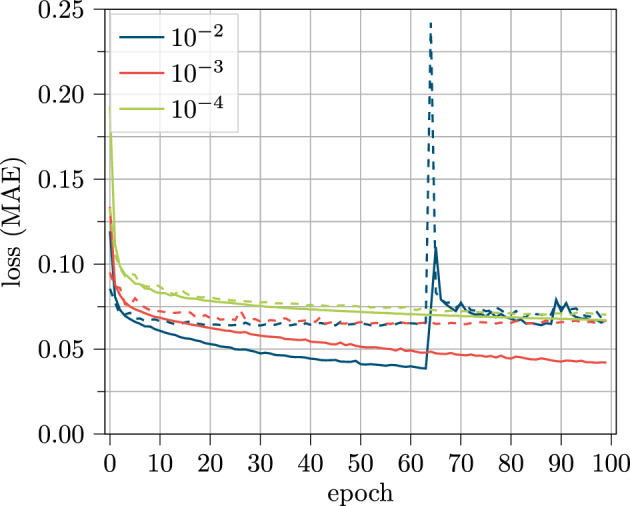
Loss curve for training (solid) and validation (dashed) data over the training process of 100 epochs for 258 LSTM cells and the sensor based feature set for different learning rates using the RM.

This assumption is further investigated in Fig. 16. Here, the loss curve for the same hyperparameters but with different learning rates is displayed.

Here it becomes clear that the training behavior of the RM is not ideal for the chosen learning rate of $10^{-2}$. Lowering the learning rate results in a more steady training behavior, but too low learning rates cause worse model results and slower training process.

As a result, the learning rate is a hyperparameter which has a high influence and should be investigated in more detail for further use of the RM model. In contrast, the other models seem to be less affected by higher learning rates. In general, for the NRM and the SNRM, a more steady training behavior can be obtained, which could be an argument in favor of these models.

In case of using the RM in the future, also adaptive learning rates could be an instrument to enhance the model behavior.

### Energy consumption

**Figure 17:**
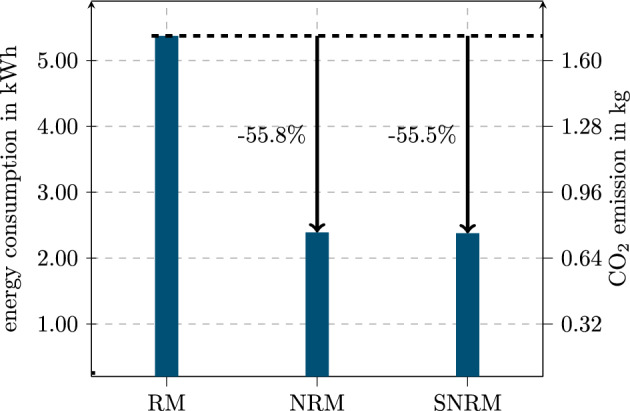
Energy consumption and CO_2_ emissions for training of the RM, NRM and SNRM for the presented grid search and k-fold cross validation.

In regard to the energy consumption of the training of the model, the relevant data for the whole training is displayed in Fig. 17. The energy consumption was estimated by multiplying the GPU’s maximum power consumption of 250 W^24^ with the model-specific training time. As the GPU was operating at full load throughout the training process, this estimate represents a realistic upper bound of the actual energy usage. Data preprocessing is not included in this calculation, but its contribution is considered negligible compared to training. While absolute runtimes reflect performance under the used software versions, relative comparisons between models are expected to remain valid across framework updates.

The CO_2_ emissions are calculated based on the average CO_2_ emissions from electricity mix in Germany between October 2023 and 2024. The data is based on^25^.

These results underlie the previous results regarding training time. The RM has more than double the energy consumption and CO_2_ emissions over the whole training compared to the NRM and SNRM. These two models have a pretty similar energy demand for the shown training process.

Even though the absolute energy consumption and CO_2_ emission presented in Fig. 17 is exemplary for the shown work, the relative values can be extrapolated for bigger grid searches, more training epochs and more training data. Depending on the application, the absolute difference can be correspondingly greater.

## Conclusion

In this work, three different LSTM based speed prediction models were evaluated for two different feature sets. For all models, a grid search was executed to evaluate the influence of different hyperparameters. The resulting best models were then compared to each other. It was found that the best models for each layout do not differ a lot regarding their prediction capability. The differences are almost negligible, but there are significant differences regarding the energy demand required to train those models.

This could be reasoned by similar capabilities of the models or by the underlying data. For this application, it is reasonable to assume that there are too few recurring patterns within a 10 s time lookback and within the dataset, so that no Markov chain can be identified. In the future, this needs to be investigated by having distinct and recurring patterns within the used feature set and using enough training data.

In contrast to all works in Sect. ”Introduction”, that are trying to use more complex models for speed predictions, this work shows that also simple models have the capability to predict vehicle speeds with a similar high accuracy as more complex models. As presented in this paper, the calculation time and consequently the energy demand is rising significantly for more complex networks. The results of this work show that incorporating computation time and energy demand does not necessarily imply poorer prediction performance. Using also this parameter for choosing a model or hyperparameter set is therefore favorable for future works.

This paper also shows that the energy demand to train a LSTM-based prediction model needs to be analyzed in order to evaluate a system’s impact regarding energy demand and CO_2_ emissions. If the benefits of e.g. an adapted operation strategy do not weigh up the impact of the implementation of the prediction model, the overall system must be scrutinised.

For future tasks of speed prediction, it is also recommended to evaluate simple models like the NRM or SNRM regarding their ability to predict vehicle speeds. This paper shows that these simple models can reduce energy demand by a lot while not losing much predictive performance.

## Data Availability

The datasets generated and analyzed during the current study are not publicly available due to their large volume and associated storage limitations, but are available from the corresponding author on reasonable request.
